# Supplementary material for: Multi-group symbiotic evolutionary mechanisms of a digital innovation ecosystem: Numerical simulation and case study
Source: PLoS One. 2024 Apr 4;19(4):e0300218. doi: 10.1371/journal.pone.0300218 (PMC10994354; doi:10.1371/journal.pone.0300218)
Supplement: S1 File — (PDF) [file pone.0300218.s001.pdf]

```
function dydt=gongsheng(t,y,r1,r2,r3,n1,n2,n3,x12,x13,x21,x23,x31,x32)
dydt=zeros(3,1);
dydt(1)= r1*y(1)*(1-y(1)/n1-x21*y(2)/n2-x31*y(3)/n3);
dydt(2)= r2*y(2)*(1-y(2)/n2-x12*y(1)/n1-x32*y(3)/n3);
dydt(3)= r3*y(3)*(1-y(3)/n3-x13*y(1)/n1-x23*y(2)/n2);
end
```

Freestanding symbiotic mode

```
clc;clear;
r1=0.05,r2=0.01,r3=0.02,n1=1000,n2=1000,n3=1000,x12=0,x13=0,x21=0,x23=0,x31=0,x32=0;
figure(1)
%%%%%% line1
[t,y]=ode45(@(t,y)
gongsheng(t,y,r1,r2,r3,n1,n2,n3,x12,x13,x21,x23,x31,x32),[0,1000],[100,100,100]);
plot(t,y(:,1),'h-
','color',[1,0.84314,0],'MarkerSize',8,'LineWidth',1,'MarkerFaceColor',[1,0.843314,0])
;
hold on
%%%%%% line2
plot(t,y(:,2),'*-.','color',[0.93333,0.5098,0.93333],'MarkerSize',8,'LineWidth',1,'Mar
kerFaceColor',[1,0.843314,0]);
hold on
%%%%%% line3
plot(t,y(:,3),'o:','color',[0,0.74902,1],'MarkerSize',8,'LineWidth',1);
hold on
set(gca,'XTick',[0:100:1000],'YTick',[0:200:1200])
axis([0 1000 0 1200])
xlabel('Symbiosis evolution time', 'interpreter','latex','FontName','Times New
Roman','FontSize',12);
ylabel('Symbiotic population size','interpreter','latex','FontName','Times New
Roman','FontSize',12);
legend('Core enterprise groups  \alpha_{ed}=0,\alpha_{ec}=0', 'Digital platform groups
\alpha_{de}=0,\alpha_{dc}=0', 'University research institution groups
\alpha_{ce}=0,\alpha_{cd}=0',  'location', 'Southeast');
set(legend,'FontName','Times New Roman','FontSize',12)
```

Competitive symbiosis mode

```
clc;clear;
figure(2)
subplot(2,2,1)
r1=0.05,r2=0.01,r3=0.02,n1=1000,n2=1000,n3=1000,x12=0.1,x13=0.2,x21=0.2,x23=0.4,x31=0.
1,x32=0.3;
%%%%%% line1
[t,y]=ode45(@(t,y)
```

```
gongsheng(t,y,r1,r2,r3,n1,n2,n3,x12,x13,x21,x23,x31,x32),[0,1000],[100,100,100]);
plot(t,y(:,1),'h-
','color',[1,0.84314,0],'MarkerSize',4,'LineWidth',1,'MarkerFaceColor',[1,0.843314,0])
;
hold on
%%%%%% line2
plot(t,y(:,2),'*-.','color',[0.93333,0.5098,0.93333],'MarkerSize',4,'LineWidth',1,'Mar
kerFaceColor',[1,0.843314,0]);
hold on
%%%%%% line3
plot(t,y(:,3),'o:','color',[0,0.74902,1],'MarkerSize',4,'LineWidth',1);
hold on
set(gca,'XTick',[0:100:1000],'YTick',[0:200:1200])
axis([0 1000 0 1200])
xlabel('Symbiosis evolution time', 'interpreter','latex','FontName','Times New
Roman','FontSize',12);
ylabel('Symbiotic population size','interpreter','latex','FontName','Times New
Roman','FontSize',12);
legend('Core enterprise groups  \alpha_{ed}=0.1,\alpha_{ec}=0.2', 'Digital platform
groups  \alpha_{de}=0.2,\alpha_{dc}=0.4', 'University research institution groups
\alpha_{ce}=0.1,\alpha_{cd}=0.3',  'location', 'Northeast');
set(legend,'FontName','Times New Roman')
title('(a)','position',[500,-300])
subplot(2,2,2)
r1=0.05,r2=0.01,r3=0.02,n1=1000,n2=1000,n3=1000,x12=0.5,x13=0.4,x21=0.3,x23=0.2,x31=0.
4,x32=0.6;
%%%%%% line1
[t,y]=ode45(@(t,y)
gongsheng(t,y,r1,r2,r3,n1,n2,n3,x12,x13,x21,x23,x31,x32),[0,1000],[100,100,100]);
plot(t,y(:,1),'h-
','color',[1,0.84314,0],'MarkerSize',4,'LineWidth',1,'MarkerFaceColor',[1,0.843314,0])
;
hold on
%%%%%% line2
plot(t,y(:,2),'*-.','color',[0.93333,0.5098,0.93333],'MarkerSize',4,'LineWidth',1,'Mar
kerFaceColor',[1,0.843314,0]);
hold on
%%%%%% line3
plot(t,y(:,3),'o:','color',[0,0.74902,1],'MarkerSize',4,'LineWidth',1);
hold on
set(gca,'XTick',[0:100:1000],'YTick',[0:200:1200])
axis([0 1000 0 1200])
xlabel('Symbiosis evolution time', 'interpreter','latex','FontName','Times New
Roman','FontSize',12);
```

```
ylabel('Symbiotic population size','interpreter','latex','FontName','Times New
Roman','FontSize',12);
legend('Core enterprise groups  \alpha_{ed}=0.5,\alpha_{ec}=0.4', 'Digital platform
groups  \alpha_{de}=0.3,\alpha_{dc}=0.2', 'University research institution groups
\alpha_{ce}=0.4,\alpha_{cd}=0.6',  'location', 'Northeast');
set(legend,'FontName','Times New Roman')
title('(b)','position',[500,-300])
subplot(2,2,3)
r1=0.05,r2=0.01,r3=0.02,n1=1000,n2=1000,n3=1000,x12=1.2,x13=1.4,x21=1.3,x23=1.5,x31=1.
4,x32=1.6;
%%%%%% line1
[t,y]=ode45(@(t,y)
gongsheng(t,y,r1,r2,r3,n1,n2,n3,x12,x13,x21,x23,x31,x32),[0,1000],[100,100,100]);
plot(t,y(:,1),'h-
','color',[1,0.84314,0],'MarkerSize',4,'LineWidth',1,'MarkerFaceColor',[1,0.843314,0])
;
hold on
%%%%%% line2
plot(t,y(:,2),'*-.','color',[0.93333,0.5098,0.93333],'MarkerSize',4,'LineWidth',1,'Mar
kerFaceColor',[1,0.843314,0]);
hold on
%%%%%% line3
plot(t,y(:,3),'o:','color',[0,0.74902,1],'MarkerSize',4,'LineWidth',1);
hold on
set(gca,'XTick',[0:100:1000],'YTick',[0:200:1200])
axis([0 1000 0 1200])
xlabel('Symbiosis evolution time', 'interpreter','latex','FontName','Times New
Roman','FontSize',12);
ylabel('Symbiotic population size','interpreter','latex','FontName','Times New
Roman','FontSize',12);
legend('Core enterprise groups   \alpha_{ed}=1.2,\alpha_{ec}=1.4', 'Digital platform
groups  \alpha_{de}=1.3,\alpha_{dc}=1.5', 'University research institution groups
\alpha_{ce}=1.4,\alpha_{cd}=1.6',  'location', 'East');
set(legend,'FontName','Times New Roman')
title('(c)','position',[500,-300])
subplot(2,2,4)
r1=0.05,r2=0.01,r3=0.02,n1=1000,n2=1000,n3=1000,x12=1.1,x13=1.2,x21=1.3,x23=1.2,x31=1.
5,x32=1.2;
%%%%%% line1
[t,y]=ode45(@(t,y)
gongsheng(t,y,r1,r2,r3,n1,n2,n3,x12,x13,x21,x23,x31,x32),[0,1000],[100,100,100]);
plot(t,y(:,1),'h-
','color',[1,0.84314,0],'MarkerSize',4,'LineWidth',1,'MarkerFaceColor',[1,0.843314,0])
;
```

```
hold on
%%%%%% line2
plot(t,y(:,2),'*-.','color',[0.93333,0.5098,0.93333],'MarkerSize',4,'LineWidth',1,'Mar
kerFaceColor',[1,0.843314,0]);
hold on
%%%%%% line3
plot(t,y(:,3),'o:','color',[0,0.74902,1],'MarkerSize',4,'LineWidth',1);
hold on
set(gca,'XTick',[0:100:1000],'YTick',[0:200:1200])
axis([0 1000 0 1200])
xlabel('Symbiosis evolution time', 'interpreter','latex','FontName','Times New
Roman','FontSize',12);
ylabel('Symbiotic population size','interpreter','latex','FontName','Times New
Roman','FontSize',12);
legend('Core enterprise groups  \alpha_{ed}=1.1,\alpha_{ec}=1.2', 'Digital platform
groups  \alpha_{de}=1.3,\alpha_{dc}=1.2', 'University research institution groups
\alpha_{ce}=1.5,\alpha_{cd}=1.2',  'location', 'Northeast');
set(legend,'FontName','Times New Roman')
title('(d)','position',[500,-300])
```

Parasitic symbiotic mode

```
clc;clear;
figure(3)
subplot(2,2,1)
r1=0.05,r2=0.01,r3=0.02,n1=1000,n2=1000,n3=1000,x12=-0.3,x13=-
0.4,x21=0.3,x23=0.1,x31=0.2,x32=0.3;
%%%%%% line1
[t,y]=ode45(@(t,y)
gongsheng(t,y,r1,r2,r3,n1,n2,n3,x12,x13,x21,x23,x31,x32),[0,1000],[100,100,100]);
plot(t,y(:,1),'h-
','color',[1,0.84314,0],'MarkerSize',4,'LineWidth',1,'MarkerFaceColor',[1,0.843314,0])
;
hold on
%%%%%% line2
plot(t,y(:,2),'*-.','color',[0.93333,0.5098,0.93333],'MarkerSize',4,'LineWidth',1,'Mar
kerFaceColor',[1,0.843314,0]);
hold on
%%%%%% line3
plot(t,y(:,3),'o:','color',[0,0.74902,1],'MarkerSize',4,'LineWidth',1);
hold on
set(gca,'XTick',[0:100:1000],'YTick',[0:200:1200])
axis([0 1000 0 1200])
xlabel('Symbiosis evolution time', 'interpreter','latex','FontName','Times New
Roman','FontSize',12);
```

```
ylabel('Symbiotic population size','interpreter','latex','FontName','Times New Roman','FontSize',12);
legend('Core enterprise groups  \alpha_{ed}=-0.3,\alpha_{ec}=-0.4', 'Digital platform groups  \alpha_{de}=0.3,\alpha_{dc}=0.1', 'University research institution groups \alpha_{ce}=0.2,\alpha_{cd}=0.3',  'location', 'Southeast');
set(legend,'FontName','Times New Roman')
title('(a)','position',[500,-300])
subplot(2,2,2)
r1=0.05,r2=0.01,r3=0.02,n1=1000,n2=1000,n3=1000,x12=0.1,x13=0.2,x21=-0.3,x23=-0.2,x31=0.4,x32=0.1;
%%%%%% line1
[t,y]=ode45(@(t,y)
gongsheng(t,y,r1,r2,r3,n1,n2,n3,x12,x13,x21,x23,x31,x32),[0,1000],[100,100,100]);
plot(t,y(:,1),'h-
','color',[1,0.84314,0],'MarkerSize',4,'LineWidth',1,'MarkerFaceColor',[1,0.843314,0])
;
hold on
%%%%%% line2
plot(t,y(:,2),'*-.','color',[0.93333,0.5098,0.93333],'MarkerSize',4,'LineWidth',1,'MarkerFaceColor',[1,0.843314,0]);
hold on
%%%%%% line3
plot(t,y(:,3),'o:','color',[0,0.74902,1],'MarkerSize',4,'LineWidth',1);
hold on
set(gca,'XTick',[0:100:1000],'YTick',[0:200:1200])
axis([0 1000 0 1200])
xlabel('Symbiosis evolution time', 'interpreter','latex','FontName','Times New Roman','FontSize',12);
ylabel('Symbiotic population size','interpreter','latex','FontName','Times New Roman','FontSize',12);
legend('Core enterprise groups  \alpha_{ed}=0.1,\alpha_{ec}=0.2', 'Digital platform groups  \alpha_{de}=-0.3,\alpha_{dc}=-0.2', 'University research institution groups \alpha_{ce}=0.4,\alpha_{cd}=0.1',  'location', 'Southeast');
set(legend,'FontName','Times New Roman')
title('(b)','position',[500,-300])
subplot(2,2,3)
r1=0.05,r2=0.01,r3=0.02,n1=1000,n2=1000,n3=1000,x12=0.2,x13=0.1,x21=0.4,x23=0.3,x31=-0.4,x32=-0.2;
%%%%%% line1
[t,y]=ode45(@(t,y)
gongsheng(t,y,r1,r2,r3,n1,n2,n3,x12,x13,x21,x23,x31,x32),[0,1000],[100,100,100]);
plot(t,y(:,1),'h-
','color',[1,0.84314,0],'MarkerSize',4,'LineWidth',1,'MarkerFaceColor',[1,0.843314,0])
;
```

```
hold on
%%%%%% line2
plot(t,y(:,2),'*-.','color',[0.93333,0.5098,0.93333],'MarkerSize',4,'LineWidth',1,'MarkerFaceColor',[1,0.843314,0]);
hold on
%%%%%% line3
plot(t,y(:,3),'o:','color',[0,0.74902,1],'MarkerSize',4,'LineWidth',1);
hold on
set(gca,'XTick',[0:100:1000],'YTick',[0:200:1200])
axis([0 1000 0 1200])
xlabel('Symbiosis evolution time', 'interpreter','latex','FontName','Times New Roman','FontSize',12);
ylabel('Symbiotic population size','interpreter','latex','FontName','Times New Roman','FontSize',12);
legend('Core enterprise groups  \alpha_{ed}=0.2,\alpha_{ec}=0.1', 'Digital platform groups  \alpha_{de}=0.4,\alpha_{dc}=0.3', 'University research institution groups \alpha_{ce}=-0.4,\alpha_{cd}=-0.2',  'location', 'SouthEast');
set(legend,'FontName','Times New Roman')
title('(c)','position',[500,-300])
subplot(2,2,4)
r1=0.05,r2=0.01,r3=0.02,n1=1000,n2=1000,n3=1000,x12=0.3,x13=0.4,x21=-0.1,x23=0.3,x31=-0.2,x32=0.3;
%%%%%% line1
[t,y]=ode45(@(t,y) gongsheng(t,y,r1,r2,r3,n1,n2,n3,x12,x13,x21,x23,x31,x32),[0,1000],[100,100,100]);
plot(t,y(:,1),'h-','color',[1,0.84314,0],'MarkerSize',4,'LineWidth',1,'MarkerFaceColor',[1,0.843314,0]);
hold on
%%%%%% line2
plot(t,y(:,2),'*-.','color',[0.93333,0.5098,0.93333],'MarkerSize',4,'LineWidth',1,'MarkerFaceColor',[1,0.843314,0]);
hold on
%%%%%% line3
plot(t,y(:,3),'o:','color',[0,0.74902,1],'MarkerSize',4,'LineWidth',1);
hold on
set(gca,'XTick',[0:100:1000],'YTick',[0:200:1200])
axis([0 1000 0 1200])
xlabel('Symbiosis evolution time', 'interpreter','latex','FontName','Times New Roman','FontSize',12);
ylabel('Symbiotic population size','interpreter','latex','FontName','Times New Roman','FontSize',12);
legend('Core enterprise groups  \alpha_{ed}=0.3,\alpha_{ec}=0.4', 'Digital platform groups  \alpha_{de}=-0.1,\alpha_{dc}=0.3', 'University research institution groups
```

```
\alpha_{ce}=-0.2,\alpha_{cd}=0.3',  'location', 'Southeast');
set(legend,'FontName','Times New Roman')
title('(d)','position',[500,-300])
```

Biased mutual benefit mode

```
clc;clear;
figure(4)
subplot(2,2,1)
r1=0.05,r2=0.01,r3=0.02,n1=1000,n2=1000,n3=1000,x12=0,x13=0,x21=-0.3,x23=0.02,x31=-
0.2,x32=0.01;
%%%%%% line1
[t,y]=ode45(@(t,y)
gongsheng(t,y,r1,r2,r3,n1,n2,n3,x12,x13,x21,x23,x31,x32),[0,1000],[100,100,100]);
plot(t,y(:,1),'h-
','color',[1,0.84314,0],'MarkerSize',4,'LineWidth',1,'MarkerFaceColor',[1,0.843314,0])
;
hold on
%%%%%% line2
plot(t,y(:,2),'*-.','color',[0.93333,0.5098,0.93333],'MarkerSize',4,'LineWidth',1,'Mar
kerFaceColor',[1,0.843314,0]);
hold on
%%%%%% line3
plot(t,y(:,3),'o:','color',[0,0.74902,1],'MarkerSize',4,'LineWidth',1);
hold on
set(gca,'XTick',[0:100:1000],'YTick',[0:200:1600])
axis([0 1000 0 1600])
xlabel('Symbiosis evolution time', 'interpreter','latex','FontName','Times New
Roman','FontSize',12);
ylabel('Symbiotic population size','interpreter','latex','FontName','Times New
Roman','FontSize',12);
legend('Core enterprise groups  \alpha_{ed}=0,\alpha_{ec}=0', 'Digital platform groups
\alpha_{de}=-0.3,\alpha_{dc}=0.02', 'University research institution groups
\alpha_{ce}=-0.2,\alpha_{cd}=0.01',  'location', 'Southeast');
set(legend,'FontName','Times New Roman')
title('(a)','position',[500,-400])
subplot(2,2,2)
r1=0.05,r2=0.01,r3=0.02,n1=1000,n2=1000,n3=1000,x12=-
0.1,x13=0.01,x21=0,x23=0,x31=0.02,x32=-0.2;
%%%%%% line1
[t,y]=ode45(@(t,y)
gongsheng(t,y,r1,r2,r3,n1,n2,n3,x12,x13,x21,x23,x31,x32),[0,1000],[100,100,100]);
plot(t,y(:,1),'h-
','color',[1,0.84314,0],'MarkerSize',4,'LineWidth',1,'MarkerFaceColor',[1,0.843314,0])
;
```

```
hold on
%%%%%% line2
plot(t,y(:,2),'*-.','color',[0.93333,0.5098,0.93333],'MarkerSize',4,'LineWidth',1,'Mar
kerFaceColor',[1,0.843314,0]);
hold on
%%%%%% line3
plot(t,y(:,3),'o:','color',[0,0.74902,1],'MarkerSize',4,'LineWidth',1);
hold on
set(gca,'XTick',[0:100:1000],'YTick',[0:200:1600])
axis([0 1000 0 1600])
xlabel('Symbiosis evolution time', 'interpreter','latex','FontName','Times New
Roman','FontSize',12);
ylabel('Symbiotic population size','interpreter','latex','FontName','Times New
Roman','FontSize',12);
legend('Core enterprise groups  \alpha_{ed}=-0.1,\alpha_{ec}=0.01', 'Digital platform
groups  \alpha_{de}=0,\alpha_{dc}=0', 'University research institution groups
\alpha_{ce}=0.02,\alpha_{cd}=-0.2',  'location', 'Southeast');
set(legend,'FontName','Times New Roman')
title('(b)','position',[500,-400])
subplot(2,2,3)
r1=0.05,r2=0.01,r3=0.02,n1=1000,n2=1000,n3=1000,x12=0.03,x13=-0.1,x21=0.01,x23=-
0.3,x31=0,x32=0;
%%%%%% line1
[t,y]=ode45(@(t,y)
gongsheng(t,y,r1,r2,r3,n1,n2,n3,x12,x13,x21,x23,x31,x32),[0,1000],[100,100,100]);
plot(t,y(:,1),'h-
','color',[1,0.84314,0],'MarkerSize',4,'LineWidth',1,'MarkerFaceColor',[1,0.843314,0])
;
hold on
%%%%%% line2
plot(t,y(:,2),'*-.','color',[0.93333,0.5098,0.93333],'MarkerSize',4,'LineWidth',1,'Mar
kerFaceColor',[1,0.843314,0]);
hold on
%%%%%% line3
plot(t,y(:,3),'o:','color',[0,0.74902,1],'MarkerSize',4,'LineWidth',1);
hold on
set(gca,'XTick',[0:100:1000],'YTick',[0:200:1600])
axis([0 1000 0 1600])
xlabel('Symbiosis evolution time', 'interpreter','latex','FontName','Times New
Roman','FontSize',12);
ylabel('Symbiotic population size','interpreter','latex','FontName','Times New
Roman','FontSize',12);
legend('Core enterprise groups   \alpha_{ed}=0.03,\alpha_{ec}=-0.1', 'Digital platform
groups  \alpha_{de}=0.01,\alpha_{dc}=-0.3', 'University research institution groups
```

```
\alpha_{ce}=0,\alpha_{cd}=0',  'location', 'Southeast');
set(legend,'FontName','Times New Roman')
title('(c)','position',[500,-400])
subplot(2,2,4)
r1=0.05,r2=0.01,r3=0.02,n1=1000,n2=1000,n3=1000,x12=-0.3,x13=-
0.4,x21=0,x23=0.2,x31=0,x32=0.1;
%%%%%% line1
[t,y]=ode45(@(t,y)
gongsheng(t,y,r1,r2,r3,n1,n2,n3,x12,x13,x21,x23,x31,x32),[0,1000],[100,100,100]);
plot(t,y(:,1),'h-
','color',[1,0.84314,0],'MarkerSize',4,'LineWidth',1,'MarkerFaceColor',[1,0.843314,0])
;
hold on
%%%%%% line2
plot(t,y(:,2),'*-.','color',[0.93333,0.5098,0.93333],'MarkerSize',4,'LineWidth',1,'Mar
kerFaceColor',[1,0.843314,0]);
hold on
%%%%%% line3
plot(t,y(:,3),'o:','color',[0,0.74902,1],'MarkerSize',4,'LineWidth',1);
hold on
set(gca,'XTick',[0:100:1000],'YTick',[0:200:1600])
axis([0 1000 0 1600])
xlabel('Symbiosis evolution time', 'interpreter','latex','FontName','Times New
Roman','FontSize',12);
ylabel('Symbiotic population size','interpreter','latex','FontName','Times New
Roman','FontSize',12);
legend('Core enterprise groups  \alpha_{ed}=-0.3,\alpha_{ec}=-0.4', 'Digital platform
groups  \alpha_{de}=0,\alpha_{dc}=0.2', 'University research institution groups
\alpha_{ce}=0,\alpha_{cd}=0.1',  'location', 'Southeast');
set(legend,'FontName','Times New Roman')
title('(d)','position',[500,-400])
```

Mutually beneficial symbiotic model

```
clc;clear;
r1=0.05,r2=0.01,r3=0.02,n1=1000,n2=1000,n3=1000,x12=-0.2,x13=-0.1,x21=-0.1,x23=-
0.2,x31=-0.1,x32=-0.3;
figure(5)
%%%%%% line1
[t,y]=ode45(@(t,y)
gongsheng(t,y,r1,r2,r3,n1,n2,n3,x12,x13,x21,x23,x31,x32),[0,1000],[100,100,100]);
plot(t,y(:,1),'h-
','color',[1,0.84314,0],'MarkerSize',8,'LineWidth',1,'MarkerFaceColor',[1,0.843314,0])
;
hold on
```

```
%%%%%% line2
plot(t,y(:,2),'*-.','color',[0.93333,0.5098,0.93333],'MarkerSize',8,'LineWidth',1,'MarkerFaceColor',[1,0.843314,0]);
hold on
%%%%%% line3
plot(t,y(:,3),'o:','color',[0,0.74902,1],'MarkerSize',8,'LineWidth',1);
hold on
set(gca,'XTick',[0:100:1000],'YTick',[0:200:1800])
axis([0 1000 0 1800])
xlabel('Symbiosis evolution time', 'interpreter','latex','FontName','Times New Roman','FontSize',12);
ylabel('Symbiotic population size','interpreter','latex','FontName','Times New Roman','FontSize',12);
legend('Core enterprise groups  \alpha_{ed}=-0.2,\alpha_{ec}=-0.1', 'Digital platform groups  \alpha_{de}=-0.1,\alpha_{dc}=-0.2', 'University research institution groups \alpha_{ce}=-0.1,\alpha_{cd}=-0.3',  'location', 'Southeast');
set(legend,'FontName','Times New Roman','FontSize',12)
```
