# Supplementary material for: Multi-group symbiotic evolutionary mechanisms of a digital innovation ecosystem: Numerical simulation and case study
Source: PLoS One. 2024 Apr 4;19(4):e0300218. doi: 10.1371/journal.pone.0300218 (PMC10994354; doi:10.1371/journal.pone.0300218)
Supplement: Attachment — Submitted filename: Response to Reviewers.docx [file pone.0300218.s002.docx]

Dear Editor and Reviewers:
 Thank you for your letter and the reviewers’ comments concerning our manuscript entitled **“Simulation study on symbiotic evolution of digital innovation ecosystems” (ID: PONE-D-23-38488)**. Those comments and suggestions are all valuable and very helpful for revising and improving our paper, as well as the essential guiding significance to our research. Based on these comments and suggestions, we have carefully modified the original manuscript. We hope the revised manuscript will meet your magazine’s standards. The revised portion is marked in yellow in this paper. The point-to-point responses to the academic editor and reviewer’s comments are listed as follows:

**Academic editor:**

**Comment 1:**

Please ensure that your manuscript meets PLOS ONE's style requirements, including those for file naming. The PLOS ONE style templates can be found at <https://journals.plos.org/plosone/s/file?id=wjVg/PLOSOne_formatting_sample_m-ain_body.pdf> and <https://journals.plos.org/plosone/s/file?id=ba62/PLOSOne_format-ting_sample_title_authors_affiliations.pdf>

**Response:**

Thank you very much for your valuable comments!

We have carefully read the journal's requirements to ensure that the revised manuscript meets the relevant requirements of PLOS ONE.

**Comment 2:**

Please note that PLOS ONE has specific guidelines on code sharing for

submissions in which author-generated code underpins the findings in the man-uscript. In these cases, all author-generated code must be made available with-out restrictions upon publication of the work. Please review our guidelines at

<https://journals.plos.org/plosone/s/materials-and-software-sharing#loc-sharing-code>

and ensure that your code is shared in a way that follows best practice and

facilitates reproducibility and reuse.

**Response:**

Thank you for your helpful comments!

This paper requires only one hardware and software platform, MATLAB, which readers commonly use. In addition, all codes involved in the simulation part of the manuscript are provided in the supporting information file **"S1_File.pdf"**.

**Comment 3:**

We suggest you thoroughly copyedit your manuscript for language usage, spelling, and grammar. If you do not know anyone who can help you do this, you may wish to consider employing a professional scientific editing service.

**Response:**

Thank you for your suggestion！

To improve the language and logic of this article, we have hired the professional editing agency: Editage. Please refer to the Editing Certificate.

**Comment 4:**

We note that the grant information you provided in the ‘Funding Information’ and ‘Financial Disclosure’ sections do not match. When you resubmit, please ensure that you provide the correct grant numbers for the awards you received for your study in the ‘Funding Information’ section.

**Response:**

Thank you for your suggestions！

We have ensured that the research grant numbers provided in the "Funding Information" section of the resubmission are correct.

**Comment 5:**

Please provide a complete Data Availability Statement in the submission form, ensuring you include all necessary access information or a reason for why you are unable to make your data freely accessible. If your research concerns only data provided within your submission, please write "All data are in the manuscript and/or supporting information files" as your Data Availability Statement.

**Response:**

Thank you for your valuable suggestion！

Our research only relates to the data provided in the manuscript, and we have stated in the Data Availability Statement that "All data are in the manuscript and/or supporting information files".

**Comment 6:**

Please upload a copy of Figure 1-6. If the figure is no longer to be included as part of the submission please remove all reference to it within the text.

**Response:**

Thank you for your helpful suggestions！

We have reuploaded a copy of **Figures 1-6** in the revision file.

Special thanks to the academic editor for your valuable comments. We hope that the revised manuscript will meet your requirements.

**Reviewers:**

**Reviewer** # **1:**

**Comment 1:**

The title does not provide a core theme of the topic.

**Response:**

Thank you for your valuable and thoughtful comments!

Inspired by the Reviewer, after considering the core themes of this paper, the topic of the paper is changed to "**Multi-group symbiotic evolutionary mechanisms of a digital innovation ecosystem: Numerical simulation and case study**".

**Comment 2:**

Please specify the source of the simulation data.

**Response:**

Thank you for your helpful suggestions！

As suggested by the Reviewer, we have further specified the source of the simulation data, and the relevant expressions can be found in **Line 334-342, Page 12.**

**Comment 3:**

The language of this manuscript needs help from native speakers.

**Response:**

Thanks so much for your valuable comments!

We are very sorry for the mistakes in this manuscript and the inconvenience they caused in your reading. To improve the language of this article, we have hired the professional editing agency: Editage. Please refer to the Editing Certificate.

**Comment 4:**

Please underscore the scientific value-added to your paper in your abstract. Your abstract should clearly state the essence of the problem you are addressing, what you did and what you found and recommend. That would help a prospective reader of the abstract to decide if they wish to read the entire article.

**Response:**

Thanks so much for your useful comments!

We have underscored the scientific value-added of this work at the end of the abstract section to make our work more transparent for the reader, and the relevant statements can be found in **Line 23-26, Page 2.**

Furthermore, to help a prospective abstract section reader decide they want to read the entire article, we clearly state the essence of the problem we are addressing, what we did and what we found and recommend, which can be found in **Line 10-23, Page 2**.

**Comment 5:**

Introduction. This a very vague statement. These sentences do not provide any information on how the concept could be conceptualized? - The Introduction should have 1) a concise but complete justification of the topic's importance both academically and practically, and 2) an explanation of the gaps both in research and practice. Please review appropriate literature in the Introduction, with the research question clearly arising from that review.

**Response:**

Thanks so much for your valuable comments!

Considering the Reviewer's suggestion, we have provided a concise but complete justification of the topic's importance, academically and practically, which can be found on **Line 29-36, Page 3** of the revised manuscript.

In addition, the relevant explanation about the gaps in research and practice can be found in **Line 49-61, Page 3**.

In the introduction, we supplement the appropriate literature to demonstrate the research question clearly in the study and to give the reader a clearer understanding of what we are studying, which can be found in **Line 37-48, Page 3** and **Line 70-73, Page 4.**

**Comment 6:**

What authors wanted to convey. Here author must build research gap following the previous studies.-The manuscript does not answer the following concerns: Why is it timeliness to explore such a study? What makes this study different from the previously published studies? Are there any similarly findings in line with the previously published studies? Are the findings different from prior academic studies that were conducted elsewhere, if any? What it requires, what are the new technologies, some recent issue highlights the importance. See the following: New Energy-Driven Construction Industry: Energy development in rural China towards clean energy system: Utilization status, co-benefit mechanism and countermeasures. Frontiers in Energy Research.

**Response:**

Thank you for the valuable reference you provided!

We have referred to the paper you have provided to add relevant content to the corresponding questions, so that readers can have a clearer understanding of the research on related issues. The literature you provided is cited as **reference [13]**, and the relevant descriptions can be found in **Line 49-73, Page 3-4**.

**Comment 7:**

There is no flow in the text. It partly depends on the lack of proofreading but also on the fact that many statements and claims are made without being followed up by a clear and logical discussion. It is especially problematic in the Introduction that brings up a number of findings from different areas without linking them together.

**Response:**

Thank you for pointing out the problem and for your valuable suggestions!

We are very sorry for the fact that many statements and claims are made without being followed up by a clear and logical discussion in this manuscript and the inconvenience they caused in your reading. We have re-examined the manuscript and modified some inappropriate descriptions as much as we can.

As suggested by the Reviewer, we have supplied related explanations linked a number of findings together from different areas, which can be found in **Lines 144-160, Page 6.**

**Comment 8:**

More importantly, the choice of the questionnaire questions should be explained in light of the theory and the prior literature on the topic. The arguments are simply relationships and causes very close to the replication of many studies dealing with the same thing. For example, what is connection of upgrading path of manufacturing enterprises from the value perspective. See the following: Developing a conceptual partner matching framework for digital green innovation of agricultural high-end equipment manufacturing system toward agriculture 5.0: A Novel Niche Field Model Combined With Fuzzy VIKOR. Frontiers in Psychology, 13, 924109.

**Response:**

Thank you for pointing out the problem and for your useful suggestions!

Inspired by the Reviewer, we have added more theoretical explanations and prior literature comments to choose the questions, which can be found in **Line 162-174, Page 6.** The literature you provided has been cited as **reference [44].**

**Comment 9:**

Methodology: Model.. I suggest authors here build your main heading on Research and data methodology. Clearly explain the model building process, and what previous studies have used similar models (model testing approach).

See the following: "Incentive Mechanism for the Development of Rural New Energy Industry: New Energy Enterprise–Village Collective Linkages considering the Quantum Entanglement and Benefit Relationship", International Journal of Energy Research. https://doi.org/10.1155/2023/1675858.

A stochastic differential game of low carbon technology sharing in collaborative innovation system of superior enterprises and inferior enterprises under uncertain environment, https://doi.org/10.1515/math-2018-0056.

**Response:**

Thank you for the valuable references you provided!

As suggested by the Reviewer, we have changed the main heading to Research and data methodology, which can be found in **Line 235, Page 8.**

In addition, we have added explanations to demonstrate the model buildingprocess, which can be found in **Lines 245-249, Page 8.**

We have referred to the paper you have provided to add relevant content to review previous studies that used similar models compared with the paper. The references you provided we cite them in **reference [52-53]**, and the relevant expressions can be found in **Line 240-245, Page 8.**

**Comment 10:**

The authors should emphasize the important role of digital technology in future research. See the following: The Interaction Mechanism and Dynamic Evolution of Digital Green Innovation in the Integrated Green Building Supply Chain. Systems 2023, 11, 122. https://doi.org/10.3390/systems11030122.

**Response:**

Thank you for the valuable reference you provided!

We have referred to the paper you provided to emphasize digital technology's important role in future research. The reference you provided we cite them in **reference [60]**, and the relevant expressions can be found in **Line 640-642, Page 20.**

**Comment 11:**

Please consider this structure for manuscript final part.

-Discussion

-Conclusion

-Managerial Implication

-Practical/Social Implications.

**Response:**

Thanks so much for your valuable comments!

As suggested by the Reviewer, we have changed this structure to the final part of the manuscript.

**Comment 12:**

Please make sure your conclusions' section underscores the scientific value-added of your paper, and/or the applicability of your findings/results. Highlight the novelty of your study. In addition to summarizing the actions taken and results, please strengthen the explanation of their significance. It is recommended to use quantitative reasoning comparing with appropriate benchmarks, especially those stemming from previous work.

**Response:**

Thanks so much for your valuable suggestions!

Considering the Reviewer's suggestion, we have underscored the applicability of our results, which can be found on **Line 585-604, Page 19** of the revised manuscript.

In addition, the relevant explanations about this paper's strengthening significance, which can be found in **Lines 606-634, Pages 19-20.**

We have highlighted our study's novelty in the conclusion section by supplementing the previous literature to compare with the quantitative reasoning benchmarks of our paper, and the relevant expressions can be found in **Lines 606-615, Pages 19.**

Special thanks to you for your good comments. We hope the revised manuscript can meet your requirements.

**Reviewer** # **2:**

**Comment 1:**

The introduction effectively outlines the focus on the symbiotic evolution of digital innovation ecosystems, setting the stage for the simulation study. However, there is a need for more explicit articulation of the research objectives and the specific research questions that the simulation aims to answer. Providing this clarity would enhance the reader's understanding of the study's purpose.

**Response:**

Thank you for your valuable comments!

Inspired by the Reviewer, we have explicitly articulated the research objectives and the specific research questions the simulation aims to answer to help the reader understand our study's purpose deeply, and the relevant expressions can be found in **Line 62-73, Page 3-4**.

**Comment 2:**

The paper briefly mentions the use of simulation but lacks a thorough justification for this methodology. Why was simulation chosen over other research methods? A more explicit discussion on the advantages and limitations of simulation in the context of studying digital innovation ecosystems would strengthen the research design. Simulation studies are heavily reliant on accurate validation and calibration processes. The paper should elaborate on how the simulation model's outputs were validated against real-world data or existing theoretical frameworks. A robust validation process enhances the reliability of simulation results. To connect innovation. See the following. Exploring the Effect of Buyer Engagement on Green Product Innovation: Empirical Evidence from Manufacturers. Business Strategy and the Environment. https://doi.org/10.1002/bse.2631.Interorganizational collaboration for innovation improvement in manufacturing: The mediating role of social performance, International journal of innovation management, https://doi.org/10.1142/S1363919620500498.

**Response:**

Thank you for the valuable references you provided!

Considering the Reviewer's suggestion, we have added a more explicit discussion on the reasons for choosing simulation as the research method and the advantages and limitations of simulation, which can be found in **Line 325-329, Page 12.**

In addition, we supplement more explanations to elaborate on the process, which is the simulation model's outputs were validated against the existing theoretical framework. The references you provided we cite them in **reference [58-59]**, and the relevant expressions can be found in **Line 329-332, Page 12.**

**Comment 3:**

While the paper mentions the simulation study's key findings, a more detailed presentation and discussion of these results are needed. How do the observed patterns or outcomes align with existing theories or expectations? A thorough analysis of the simulation results would contribute to the paper's significance and impact. The paper could benefit from a discussion of the practical implications of the simulation findings. How can stakeholders in digital innovation ecosystems apply the insights derived from the simulation to enhance collaboration, innovation, or sustainability? This discussion will enhance the paper's relevance to practitioners and policymakers. The paper needs a more robust integration with existing literature on digital innovation ecosystems. How do the simulation findings align with or challenge existing theories or empirical studies in this field? A comparative analysis would provide a richer context for interpreting the simulation results.

**Response:**

Thank you for your valuable suggestions!

As suggested by the Reviewer, we have provided a more detailed presentation and discussion of these results to explore how observed patterns align with existing theories, which can be found on **Line 549-560, Page 18** of the revised manuscript.

In addition, We build on existing theoretical frameworks and refer to relevant papers, complementing the numerical simulation results primarily with case studies, and we address the practical implications of the actual cases for the simulation results in the discussion section and suggest ways to enhance digital innovation collaboration among stakeholders within the digital innovation ecosystem in response to the case findings. Therefore, we have added the relevant discussion about the practical implications of the simulation findings to explain how stakeholders in digital innovation ecosystems apply the insights derived from the simulation to enhance collaboration, innovation, or sustainability, which can be found in **Line 561-573, Page 18**.

In the discussion, we supplement existing literature on digital innovation ecosystems to demonstrate how the simulation findings align with existing theories in this field by giving a comparative analysis, which can be found in **Line 574-583, Page 18-19.**

**Comment 4:**

The conclusion should include a section on future research directions. Identifying potential areas for further exploration based on the simulation study's limitations or unanswered questions would contribute to the ongoing scholarly conversation on digital innovation ecosystems.

**Response:**

Thank you for your helpful comments!

Considering the Reviewer's suggestion, we have added relevant comments to explain potential areas for further exploration based on the simulation study's limitations or unanswered questions to contribute to the ongoing scholarly conversation on digital innovation ecosystems, which can be found in **Line 635-642, Page 20.**

**Comment 5:**

The paper is generally well-written, but attention should be given to clarity and readability. Complex terms or concepts should be explained, and the logical flow of the paper should be enhanced for easier comprehension. In summary, while the paper addresses an intriguing topic, improvements in articulating research objectives, justifying the simulation methodology, detailing model development, validating and calibrating the simulation, presenting key findings, discussing practical implications, comparing with existing literature, suggesting future research directions, and enhancing clarity would significantly strengthen the overall quality of the manuscript.

**Response:**

We are honored that our research has been highly recognized by the Reviewer, and thank you for your valuable and thoughtful comments!

As suggested by the Reviewer, we have hired a professional editing agency to improve the clarity and readability of our paper. Please refer to the Editing Certificate.

In addition, we have re-examined the manuscript, explained some complex terms or concepts, and enhanced the logical flow of the paper by providing easier comprehension as much as possible.

Following the consolidated comments of the Reviewer, we have revised the manuscript as much as we can by articulating research objectives, justifying the simulation methodology, detailing model development, validating and calibrating the simulation, presenting key findings, discussing practical implications, comparing with existing literature, suggesting future research directions, and enhancing clarity, which can be found in the previous responses.

We sincerely appreciate your recognition of our work and the suggestions you offered us, which play an essential role in improving the quality of the paper. We hope the revised manuscript can meet your requirements.

Yours Sincerely,

Liping Wu

School of Economics Management and Law

University of South China

Hengyang 421001 China

E-mail: 19907344375@163.com

February 2, 2024
